# Comparison of Venous and Capillary Differential Leukocyte Counts Using a Standard Hematology Analyzer and a Novel Microfluidic Impedance Cytometer

**DOI:** 10.1371/journal.pone.0043702

**Published:** 2012-09-20

**Authors:** Veronica S. Hollis, Judith A. Holloway, Scott Harris, Daniel Spencer, Cees van Berkel, Hywel Morgan

**Affiliations:** 1 Electronics and Computer Science, Faculty of Physical and Applied Sciences, University of Southampton, Southampton, United Kingdom; 2 Clinical and Experimental Sciences, Faculty of Medicine, University of Southampton, Southampton, United Kingdom; 3 Primary Care and Population Sciences, Faculty of Medicine, University of Southampton, Southampton, United Kingdom; 4 Philips Research United Kingdom, Cambridge, United Kingdom; Emory University/Georgia Insititute of Technology, United States of America

## Abstract

Capillary blood sampling has been identified as a potentially suitable technique for use in diagnostic testing of the full blood count (FBC) at the point-of-care (POC), for which a recent need has been highlighted. In this study we assess the accuracy of capillary blood counts and evaluate the potential of a miniaturized cytometer developed for POC testing. Differential leukocyte counts in the normal clinical range from fingerprick (capillary) and venous blood samples were measured and compared using a standard hematology analyzer. The accuracy of our novel microfluidic impedance cytometer (MIC) was then tested by comparing same-site measurements to those obtained with the standard analyzer. The concordance between measurements of fingerprick and venous blood samples using the standard hematology analyzer was high, with no clinically relevant differences observed between the mean differential leukocyte counts. Concordance data between the MIC and the standard analyzer on same-site measurements presented significantly lower leukocyte counts determined by the MIC. This systematic undercount was consistent across the measured (normal) concentration range, suggesting that an internal correction factor could be applied. Differential leukocyte counts obtained from fingerprick samples accurately reflect those from venous blood, which confirms the potential of capillary blood sampling for POC testing of the FBC. Furthermore, the MIC device demonstrated here presents a realistic technology for the future development of FBC and related tests for use at the site of patient care.

## Introduction

Point-of-care (POC) testing refers to clinical monitoring at the site of patient care, be that at the hospital bedside, GP's surgery, mobile clinic or even the patient's home. In the field of hematology, recent guidelines issued by both the British Committee for Standards in Haematology (BCSH) [1] and the International Council for Standardization in Hematology (ICSH) [2] have identified a need for POC testing of the full blood count (FBC), one of the most commonly requested indicators of patient health. For the FBC measurement, blood is usually collected by venipuncture. Disadvantages of the venipuncture method are that it requires a trained phlebotomist, generates biological waste and can cause significant discomfort to the patient. In addition, it can be difficult to obtain blood by venipuncture from the elderly, infants and young children, or very sick patients. An alternative to venipuncture is the collection of capillary blood from a skin puncture to the finger (or the heel in the case of infants) using a lancet device. The ‘fingerprick’ blood collection method more closely meets the requirements of a POC test as highlighted by the ICSH [2], since it is minimally invasive and relatively simple to perform, with basic training. It is particularly well-suited to situations in which patients require regular FBC monitoring but skilled healthcare professionals may be scarce, such as HIV/AIDS clinics in the developing world [3], [4]. However, a number of studies have reported significant differences in hematological parameters obtained from venous and capillary blood samples when using automated hematology analyzers [4]–[10]. It is therefore necessary to establish if there is a significant difference between differential leukocyte counts obtained from venous and fingerprick (capillary) blood samples. We address this issue here in healthy individuals, whose leukocyte counts are in the normal clinical range. This is an important step in assessing the potential suitability of developing a system for FBC monitoring at the site of patient care based on fingerprick blood sampling.

To meet this need for a POC blood analysis system, we are currently developing a portable, miniaturized cytometer capable of measuring the FBC from a fingerprick of blood. The cytometer is based on electrical impedance measurements of single cells flowing between electrodes positioned within a microfluidic channel, hence the device has been termed a microfluidic impedance cytometer (MIC) [11]–[13]. In this study the MIC is used to enumerate leukocyte counts only, although it is additionally capable of monitoring erythrocyte, platelet and hemoglobin concentrations [14]. Moreover, sample preparation has been performed manually, in order to focus on investigating the suitability of the device in terms of its accurate measurement of leukocytes for POC testing. Elsewhere, the use of microfluidic techniques for on-chip sample processing by the MIC has been investigated [15]. This study will determine the concordance between the MIC and the reference, a laboratory-based hematology analyzer, in measuring a 3-part leukocyte differential count, one important component of the FBC. We have previously published work in which the MIC has been used to determine relative (percentage) counts for the three main leukocyte populations [13]–[15]. In this publication we measure and compare absolute blood cell concentrations.

## Materials and Methods

### Blood sample collection

Ethical approval for this study was given by the Isle of Wight, Portsmouth and South East Hampshire Local Research Ethics Committee (Ref: 06/Q1701/137), and written informed consent was obtained from all participants. Venous samples were drawn from the antecubital fossa of the elbow into 3.5 ml Vacutainer tubes (Becton Dickinson, Oxon UK) with EDTA-K3 for anticoagulation. Fingerprick samples were obtained using a standard protocol. Blood flow to the fingertip was encouraged by placing the hand into warm water (37–38°C) for approximately 5 minutes. The fingertip skin was then punctured using a safety lancet (Sarstedt Extra) and the first drop of blood discarded to minimize excess tissue fluids [4]. Aliquots of blood from subsequent drops were then pipetted into a 0.5 ml Minicollect tube (Becton Dickinson) with EDTA-K3 for anticoagulation, giving a minimum of 0.25 ml blood. If necessary, the hand was gently massaged downwards towards the puncture site in order to obtain the required volume. Both venous and fingerprick blood samples were then aliquotted into two new tubes, one for each measurement system, and placed on blood rollers at room temperature pending measurement. All measurements were performed within 8 hours of blood sample collection.

### Sysmex XE-2100 measurement

The Sysmex XE-2100 laboratory-based hematology analyzer (Sysmex, Milton Keynes UK), based in the Hematology/Coagulation Department, Southampton University Hospitals NHS Trust, was used as a reference against which to compare the MIC. The venous and fingerprick samples from each donor were measured consecutively by an NHS operator, using at least the minimum required volume (0.175 ml) of anticoagulated blood. Samples in which any parameters were flagged by the Sysmex XE-2100 were excluded from the study.

### Blood sample preparation

Prior to measurement in the MIC (and conventional flow cytometer)blood samples were lysed on the bench at room temperature using a saponin/formic acid lytic reagent [16] in order to remove the erythrocytes, which typically outnumber the leukocytes by a factor of 1000. The lytic reagent is designed not only to lyse the erythrocytes, but also to sufficiently alter the structure of the monocyte cell membrane to allow their distinction from the similarly-sized granulocytes, whilst preserving cell integrity. To an aliquot of 50 µl blood, 600 µl of lytic reagent containing 0.05% w/v saponin and 0.12% v/v formic acid was added and agitated using a pipette for 6 seconds. The reaction was then halted by the immediate addition of 265 µl of isosmotic quench, containing 3% w/v sodium chloride and 0.6% w/v sodium carbonate, to return the blood lysate to normal osmolality (∼300 mOsm/kg H_2_O) and pH (∼6–7).

### Conventional flow cytometry

The lysed blood sample was also measured by a conventional fluorescence-based flow cytometer (FACSAria, Becton Dickinson) immediately prior to measurement by the MIC. In the event that the lysis procedure did not allow the three main leukocyte populations (granulocytes, lymphocytes and monocytes) to be resolved by the FACSAria, or showed considerable numbers of unlysed erythrocytes, the lysed sample was discarded and the procedure repeated for the same blood sample using a fresh aliquot. The absolute counts for total and individual leukocyte populations were determined using Trucount tubes (Becton Dickinson), containing a lyophilized pellet that dissolves in the sample releasing a known fixed amount of fluorescent beads. Blood lysate was added to a Trucount tube and gently mixed by vortex prior to measurement by the FACSAria. The absolute leukocyte counts for each population were determined from the Trucount bead number as specified by the manufacturers.

### MIC system

**Figure 1:**
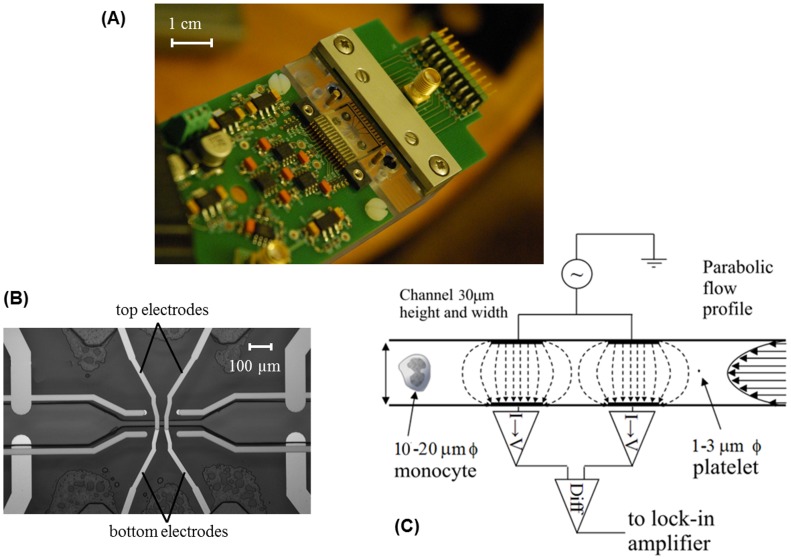
Microfluidic impedance cytometer system. Microfluidic impedance cytometer (A) front-end electronics board mounted with impedance chip (B) close-up (top view) of chip showing two pairs of overlapping electrodes above and below micro-channel (C) schematic diagram (side view) showing detection electronics and cell/platelet inside micro-channel.

The MIC system shown in Fig. 1 has been described in detail elsewhere [11]–[13]. In brief, the system consists of a circuit board onto which the microfabricated glass impedance microchip is mounted and clamped into place to maintain electrical and fluidic connections (Fig. 1 (A)). The sample is injected into a small (∼250 µl) reservoir above the inlet to the chip and drawn through the micro-channels of the chip and into a 1 ml syringe using an electronic syringe pump (Harvard Pump 11, Harvard Apparatus, MA USA). Two pairs of electrodes overlap above and below the narrowest (∼30 µm^2^) section of the micro-channel (Fig. 1 (B)). AC voltages (8 V peak-to-peak) at two frequencies (500 kHz and 2 MHz) are applied to the upper most electrode pair by a signal generator (TTi TGA 12104, Thurlby Thandar Instruments, Cambs UK). The differential current obtained as a cell or particle passes through the electrode region is measured by sensing electronics on the circuit board connected to the lower electrode pair (see Fig. 1 (C)) and two lock-in amplifiers (SR844, Stanford Research Systems, CA USA).

### MIC measurement

Once prepared, blood lysate (∼100 µl) was placed into the reservoir above the inlet of the MIC and drawn through the device at a volume flow rate of 10 µl/min, corresponding to a cell velocity through the detection region of ∼0.2 m/sec. In between sample measurements the MIC was cleaned with dH_2_O, 4M sodium hydroxide and 10% (v/v) sodium hypochlorite (bleach). The chip was then flushed with isotonic PBS containing 2 mM EDTA and 0.5% BSA to help prevent cells clumping or adhering to the internal surfaces of the MIC.

Data from the MIC was then processed to obtain measurements of electrical cell volume (*Φ*) and the opacity (*O*), or cell membrane capacitance, and analyzed using in-house software to determine absolute leukocyte (granulocyte, lymphocyte and monocyte) concentrations. The identification of the three populations was previously confirmed by fluorescent labeling of the cells [13] with monoclonal surface antibodies [17].

### Data analysis

MIC data was analyzed using two custom-written software packages. The first application, written in MATLAB (MathWorks, Cambs UK), analyses each individual event (above a noise threshold) and fits a pair of anti-symmetric Gaussian peaks to the positive and negative signals generated when a cell or particle passes through the detection electrodes [18]. The correlation coefficient (R^2^) for the fit is used to filter out poorly fitted events. The electrical volume (*Φ*, cell size) and the opacity (*O*, cell membrane capacitance) are then calculated from the amplitudes of the fitted events at both frequencies. The second application (DanMAS), written in LabView (National Instruments, Berks UK), plots the calculated electrical volume against opacity for further analysis, such as gating and counting of cell populations. Absolute counts for the three main leukocyte populations were determined using the DanMAS software as follows:
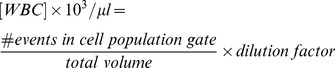
where the total volume is determined from the volume flow rate multiplied by the total time and the dilution factor is 18.3.

Linear regression was used to assess the overall correlation between the site- or device-specific assays. Concordance data was evaluated using Bland-Altman analysis, a method used to analyze the agreement between two assays or compare a new measurement technique with a reference [19]. For the fingerprick/venous and MIC/Sysmex concordances the mean of each sample pair is plotted against its difference. The mean difference is termed the bias and the limits of agreement (LOA) are defined as the bias ±1.96 standard deviations (SD). The LOA define an interval that will contain 95% of the differences between the two assays. Student's paired samples t-test was performed to evaluate the statistical significance of any differences between sample sites or devices. P values less than 0.05 were deemed as statistically significant.

## Results

### Venous-fingerprick concordance

In order to establish whether a difference exists between WBC counts obtained from venous and fingerprick blood, a concordance study was performed in which paired venous and fingerprick blood samples were taken from volunteers and measured using the Sysmex XE-2100 hematology analyzer.

**Figure 2:**
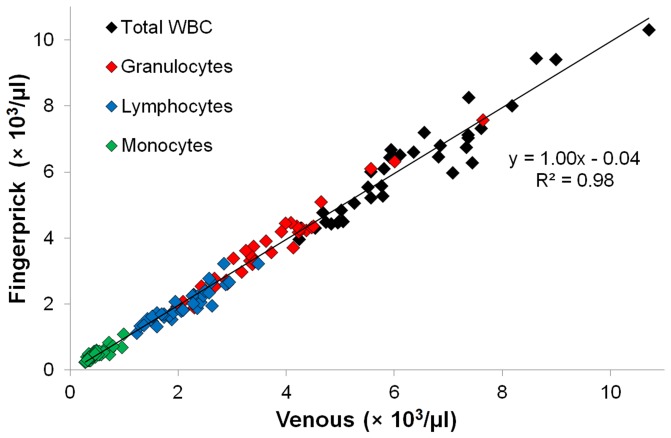
Concordance between venous and fingerprick samples on standard hematology analyzer. Concordance between venous and fingerprick blood samples for total and 3-part differential leukocyte concentrations measured using a Sysmex XE-2100 hematology analyzer (n = 36). Solid line shows linear least-squares regression of all leukocyte populations.

Fig. 2 shows the results of the concordance study, plotted as venous against fingerprick leukocyte counts for total WBC, granulocyte, lymphocyte, and monocyte concentrations (n = 36), with an R^2^ of 0.98. The granulocyte count was calculated as the sum of the neutrophil, eosinophil, and basophil counts measured by the Sysmex XE-2100, in order to obtain a 3-part differential for subsequent comparison to the MIC.

**Figure 3:**
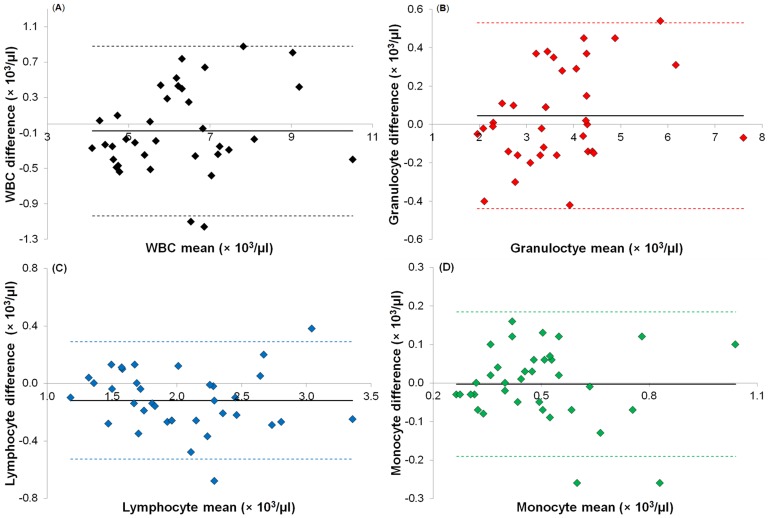
Bland-Altman analysis of venous/fingerprick concordance. Bland-Altman plots showing mean of against difference between venous and fingerprick blood samples for (A) Total WBC (B) Granulocyte (C) Lymphocyte and (D) Monocyte concentrations measured using the Sysmex XE-2100 (n = 36). Thick black line shows bias (average difference) between venous and fingerprick, dashed (colored) lines show the 95% limits of agreement (LOA), (average difference ±1.96 SD).

The data in Fig. 2 are displayed as four separate Bland-Altman plots in Fig. 3 (A)–(D), for the total WBC, granulocyte, lymphocyte and monocyte counts, respectively, where the mean of each fingerprick/venous pair is plotted against its difference. The bias (mean difference) is given by the thick black line in each plot and the LOA (95% interval) are given by the dashed colored lines.

Table 1 summarizes the Bland-Altman parameters (range, bias, and LOA) and includes the ‘normal’ clinical range [20] for each leukocyte population. For all populations the fingerprick-venous differences appear randomly distributed about the bias, with no obvious trends over the concentration range of interest. For the total leukocyte, granulocyte, and monocyte populations the bias values are close to zero (<2% from the mean venous value). The lymphocytes have a slightly larger bias (−6% from the mean venous value) and a paired t-test comparing fingerprick and venous blood samples showed a significant difference (P = 0.002) for the lymphocyte count. For comparison the mean ± SD of repeated measurements of a single venous blood sample (n = 6) by the Sysmex XE-100 are included for each population. The coefficient of variation (CV) was less than 4% for each population.

**Table 1:** Bland-Altman parameters for venous/fingerprick concordance on standard analyzer.

Leukocyte	Normal clinical range (×10^3^/µl)	Measured range (×10^3^/µl)	Bias ± SD (×10^3^/µl)	Bias percentage from mean venous count (%)	95% LOA (×10^3^/µl)	LOA percentage from mean venous count (%)	Repeatability mean ± SD (×10^3^/µl)	CV for repeatability (%)
**Total WBC**	4.0–11.0	4.11–10.51	−0.08±0.49	−1.2	0.88, −1.04	±15.3	5.69±0.15	2.6
**Granulocytes**	2.5–8.0	1.96–7.61	0.05±0.25	+1.2	0.53, −0.44	±13.4	3.34±0.11	3.3
**Lymphocytes**	1.5–3.5	1.18–3.36	−0.12±0.21	−5.6	0.29, −0.53	±19.6	1.77±0.07	4.0
**Monocytes**	0.2–0.8	0.27–1.04	0.00±0.10	−0.6	0.18, −0.19	±40.0	0.57±0.02	3.5

Bland-Altman parameters (range, bias, 95% LOA) comparing leukocyte concentrations in venous and fingerprick blood samples measured by the Sysmex XE-2100 (n = 36). For comparison the normal clinical ranges are given [20]. The repeatability (mean ± SD) for multiple measurements of a single venous sample by the Sysmex is also included (n = 6).

### Sysmex-MIC concordance

**Figure 4:**
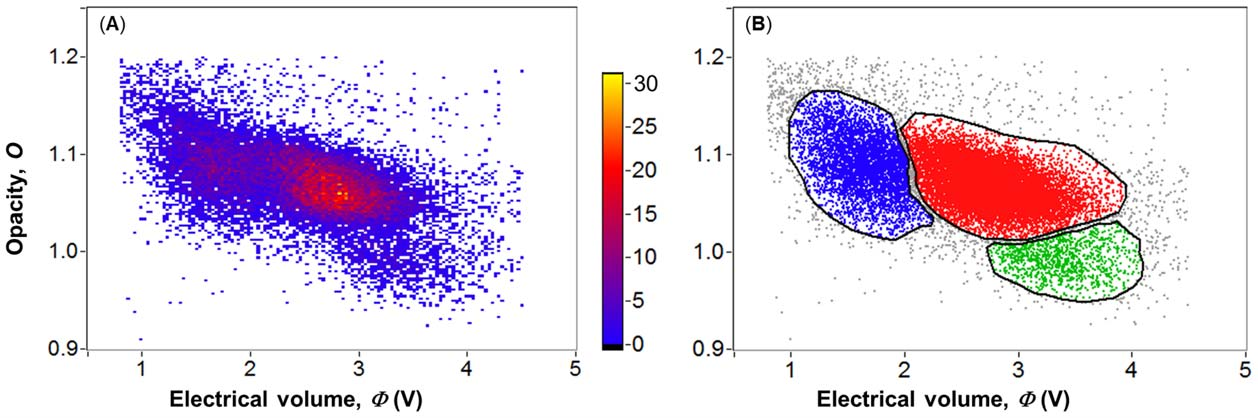
MIC impedance data measurement of venous sample. Impedance data obtained from MIC measurement of a typical venous blood sample after red cell lysis plotted as electrical cell volume, *Φ*, against opacity, *O*, displayed as (A) intensity scatter plot (color indicates cell number according to color bar) and (B) scatter plot showing manual gates (black polygons) around the three main leukocyte populations to obtain granulocyte (red), lymphocyte (blue) and monocyte (green) counts.

A selection of the paired venous and fingerprick samples collected from the total study were also measured using the MIC. Fig. 4 shows the MIC data for a typical venous blood sample plotted as electrical cell volume (*Φ*) against opacity (*O*). In Fig. 4 (A) the data is shown as an intensity scatter plot, the cell number indicated by the color bar to the right of the plot. The same data is plotted in Fig. 4 (B) and shows the polygons drawn around the populations using our custom-written DanMAS software to delineate the boundaries of and obtain counts for the granulocytes (red), lymphocytes (blue) and monocytes (green).

To determine whether or not leukocytes had been damaged by the erythrocyte lysis procedure all samples were measured by the FACSAria prior to measurement on the MIC. By comparison with same-site measurements made using the Sysmex XE-2100, the bias (and LOA) for the total WBC was 0.37 (1.57, −0.83)×10^3^/µl for the venous samples and 0.11 (1.30, −1.08)×10^3^/µl for the fingerprick samples (n = 9, data not shown). In terms of percentages of the mean (same-site) Sysmex total leukocyte counts the bias values are within 7 and 2% and the LOA widths are within ±22 and ±23% for the venous and fingerprick samples, respectively. For comparison, the CV of repeated measurements of total WBC by the FACSAria on a single venous sample was 7% (n = 4, data not shown). No significant differences were found using a paired t-test between total WBC measured by the FACSAria or the Sysmex on either venous or fingerprick samples.

**Figure 5:**
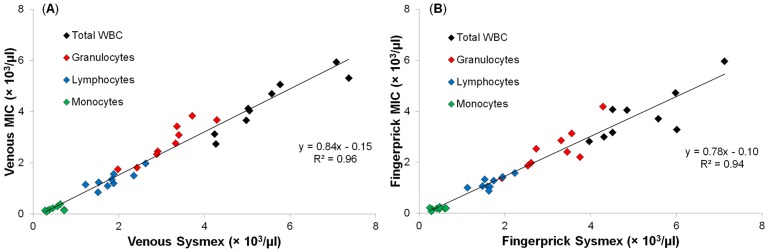
Concordance between same-site samples measured on standard analyzer and MIC. Concordance between Sysmex XE-2100 hematology analyzer and MIC for (A) venous (n = 9) and (B) fingerprick (n = 9) blood samples for total and 3-part differential leukocyte concentrations. Solid black lines show linear least-squares regression to all leukocyte populations, with first-order and correlation coefficients stated.

Fig. 5 shows the concordance between total and differential leukocytes counts measured on same-site samples using the MIC and the Sysmex for (A) venous and (B) fingerprick samples (n = 9). The Bland-Altman parameters are shown in Tables 2 and 3 for venous and fingerprick blood samples, respectively (n = 9). For all populations lower counts are obtained using the MIC, in comparison to the Sysmex, for both venous and fingerprick samples. The mean and SD of repeated measurements (n = 4) of a single venous sample by the MIC are shown in Table 2.

**Table 2:** Bland-Altman parameters for concordance between venous samples measured on standard analyzer and MIC.

Leukocyte	Measured range (×10^3^/µl)	Bias ± SD (×10^3^/µl)	Bias percentage from mean Sysmex count (%)	95% LOA (×10^3^/µl)	LOA percentage from mean Sysmex count (%)	Repeatability mean ± SD (×10^3^/µl)	CV for repeatability (%)
**Total WBC**	3.51–6.51	−1.18±0.41	−21.5	−0.38, −1.98	±14.7	4.84±0.23	4.8
**Granulocytes**	1.86–3.98	−0.35±0.28	−11.1	0.20, −0.91	±17.4	3.38±0.24	7.1
**Lymphocytes**	1.19–2.30	−0.52±0.24	−28.3	−0.05, −1.00	±25.6	1.20±0.10	8.3
**Monocytes**	0.20–0.51	−0.31±0.17	−63.2	0.02, −0.63	±68.0	0.25±0.04	16.0

Bland-Altman parameters (range, bias, 95% LOA) comparing leukocyte concentrations in venous blood samples measured by the Sysmex XE-2100 and the MIC (n = 9). For comparison the repeatability (mean ± SD) for multiple measurements by the MIC of a single venous sample is included (n = 4).

**Table 3:** Bland-Altman parameters for concordance between fingerprick samples measured on standard analyzer and MIC.

Leukocyte	Measured range (×10^3^/µl)	Bias ± SD (×10^3^/µl)	Bias percentage from mean Sysmex count (%)	95% LOA (×10^3^/µl)	LOA percentage from mean Sysmex count (%)
**Total WBC**	3.39–6.54	−1.34±0.65	−25.8	−0.07, −2.62	±24.5
**Granulocytes**	1.65–4.24	−0.63±0.44	−20.1	0.24, −1.49	±27.6
**Lymphocytes**	1.07–1.91	−0.47±0.20	−28.3	−0.08, −0.87	±23.6
**Monocytes**	0.18–0.42	−0.24±0.12	−58.5	0.00, −0.49	±57.4

Bland-Altman parameters (range, bias, 95% LOA) comparing leukocyte concentrations in fingerprick blood samples measured by the Sysmex XE-2100 and the MIC (n = 9).

## Discussion

A clinical need exists for low-cost and compact blood cell counting devices that can be deployed at the point-of-care using small volume capillary blood samples, in particular in situations where trained health care specialists are less available to extract venous blood. As part of a larger programme to develop a diagnostic device capable of measuring a 3-part differential leukocyte count from a fingerprick of blood, the primary aim of this study was to determine if fingerprick and venous blood samples give equivalent measurements.

### Concordance between venous and capillary counts

We show that in the normal clinical range a high concordance and correlation exists between absolute leukocyte counts from fingerprick and venous blood samples measured using a laboratory-based hematology analyzer. Small (not significant) bias values exist for the total WBC, granulocyte and monocyte counts, suggesting that the two blood sampling methods are essentially interchangeable for these healthy populations, whereas a significantly lower lymphocyte count was observed in the fingerprick samples. Interestingly, all other studies that have reported significant differences between leukocyte counts observed higher counts in the capillary samples [4]–[10]. Daae *et al* [5] observed significantly larger leukocyte counts in capillary versus venous blood samples, which they attributed to the fact that blood sampled by the fingertip lancet method employed was predominantly arteriolar, rather than capillary, with the larger granulocytes and monocytes becoming more concentrated along the center of fast-flowing arterioles. Yang *et al* [7] observed increased total WBC (+9.2%) and large leukocyte counts (+12.6%) counts in fingertip compared to venous samples. However, they observed a significant decrease between the first and third fingertip aliquots (20 µl) in both total WBC (−4.7%) and small leukocyte (−10.5%) counts.. They attributed these differences to a decreased accumulation of leukocytes around the fingerprick site with repeated squeezing of the fingertip. This could explain our observation of a decrease in lymphocyte counts in fingerprick compared to venous blood samples, due to excessive squeezing of the fingertip to obtain the large volume (∼250 µl) required to perform paired measurements on the Sysmex (175 µl) and the MIC (50 µl).

In addition to the bias value the width of the LOA interval is also an important factor in deciding whether differences between results obtained from different sampling methods or assays are clinically relevant, hence whether or not one assay can be used in place of another. The UK National External Quality Assessment Scheme (NEQAS) for hematology stipulates that in order to be clinically reliable, 95% of total leukocyte counts obtained by a given method or instrument should be within 8–10% of target (reference) values [21]. In the US, the Clinical Laboratory Improvement Amendments of 1988 (CLIA-88), governed by the FDA (http://www.fda.gov/), sets the standards for *in vitro* diagnostic devices, which must obtain a CLIA-88 Certificate of Waiver in order to be marketed as a self-testing POC device. The CLIA-88 regulations stipulate that measurements of total leukocyte counts must fall within 15% of reference values [22]. In our study the width of the LOA for total WBC is ±15% when compared to the mean venous value (6.3×10^3^/µl), which is outside the UK NEQAS target but within the CLIA-88 stipulation for leukocytes. This and the larger interval widths for the differential leukocyte counts are unlikely to be due to device measurement variability alone, since repeated venous measurements gave a CV (1 SD) of <4% for all leukocyte populations. Instead it may be attributed to variations in the ease of fingerprick blood sampling between donors, some requiring more or less ‘milking’ of the site to obtain the necessary blood volume to measure each sample on both devices, which could affect the differential (and hence total) leukocyte populations as described by Daae *et al.* [5] and Yang *et al* [7].

### Concordance between MIC and Sysmex

It was important when comparing the MIC data with that of the Sysmex to confirm that no leukocytes were damaged by the manual lysis of the blood sample performed in preparation for the MIC measurements. To this end, prior to measurement in the MIC, both venous and fingerprick blood samples were lysed and measured on a FACSAria flow cytometer. No significant differences were observed when comparing to leukocyte counts measured by the Sysmex analyzer, for either venous or fingerprick blood samples, suggesting that no leukocytes were lost during the erythrocyte lysis procedure. The slightly larger bias values and LOA widths obtained, in comparison with the venous-fingerprick concordance data performed on the Sysmex alone, are likely to reflect sample-to-sample variations due to the manual nature of the erythrocyte lysis. The larger CV for repeated measurements by the FACSAria of total (venous) WBC on a single sample also indicates this.

To confirm the suitability of the MIC as a POC diagnostic device, concordance between the Sysmex XE-2100 hematology analyzer and the MIC, comparing same-site blood sampling methods, was performed. Results indicate that for all samples, total and differential leukocyte counts obtained by MIC are significantly lower than those obtained by the Sysmex XE-100. Overall the negative bias values and LOA widths are slightly larger in the fingerprick compared to the venous samples. These negative bias values have no skew, or concentration-dependent trend, therefore a correction factor (or internal calibration) could be added to the analysis. Zandecki *et al* [23] noted that agglutination of leukocytes in EDTA-anticoagulated blood samples can give rise to spuriously low WBC counts in a time-dependent manner. Since the MIC measurements were performed last, meaning that the samples had the longest time in EDTA, it is possible that this effect could be contributing to the systematic bias in the MIC leukocyte counts. Moreover, the LOA widths are no greater than for those obtained for the comparison between the FACSAria and Sysmex XE-2100 measurements. This suggests that, as with the FACSAria, the erythrocyte lysis procedure is contributing predominantly to the large interval widths, rather than the device itself.

### Conclusion

In summary, we have shown that there are no clinically relevant differences (according to CLIA-88 regulations) between absolute leukocyte counts in healthy adult donors obtained from venous or fingerprick blood samples, measured on a standard automated hematology analyzer. This work demonstrates that the MIC can measure a 3-part differential leukocyte count on (manually processed) capillary blood samples. It is therefore appropriate to develop a POC device based on capillary blood sampling. Future studies with our device should focus on concordance between venous samples measured by the Sysmex XE-2100 and fingerprick samples measured by the MIC, which only requires a single drop (10–20 µl) of capillary blood, thus avoiding issues associated with fingertip squeezing for larger volumes. Further development of this system to allow on-chip sample processing could present a realistic technology for FBC and related tests at the site of patient care.
